# Spectroscopical Investigations on the Redox Chemistry of [FeFe]-Hydrogenases in the Presence of Carbon Monoxide

**DOI:** 10.3390/molecules23071669

**Published:** 2018-07-09

**Authors:** Konstantin Laun, Stefan Mebs, Jifu Duan, Florian Wittkamp, Ulf-Peter Apfel, Thomas Happe, Martin Winkler, Michael Haumann, Sven T. Stripp

**Affiliations:** 1 Department of Physics, Experimental Molecular Biophysics, Freie Universität Berlin, 14195 Berlin, Germany; konstantinlaun@aol.com; 2 Department of Physics, Biophysics of Metalloenzymes, Freie Universität Berlin, 14195 Berlin, Germany; stefan.mebs@fu-berlin.de; 3 Faculty of Biology and Biotechnology, Photobiotechnology, Ruhr-Universität Bochum, 44801 Bochum, Germany; Jifu.Duan@ruhr-uni-bochum.de (J.D.); thomas.happe@rub.de (T.H.); W.Baskerville@gmx.net (M.W.); 4 Faculty of Chemistry and Biochemistry, Inorganic Chemistry I, Ruhr-Universität Bochum, 44801 Bochum, Germany; florian.wittkamp@rub.de (F.W.); ulf.apfel@rub.de (U.-P.A.); 5 Fraunhofer UMSICHT, Osterfelder Straße 3, 46047 Oberhausen, Germany

**Keywords:** metalloenzymes, FTIR spectro-electrochemistry, hydrogenases

## Abstract

[FeFe]-hydrogenases efficiently catalyzes hydrogen conversion at a unique [4Fe–4S]-[FeFe] cofactor, the so-called H-cluster. The catalytic reaction occurs at the diiron site, while the [4Fe–4S] cluster functions as a redox shuttle. In the oxidized resting state (Hox), the iron ions of the diiron site bind one cyanide (CN^−^) and carbon monoxide (CO) ligand each and a third carbonyl can be found in the Fe–Fe bridging position (*µ*CO). In the presence of exogenous CO, A fourth CO ligand binds at the diiron site to form the oxidized, CO-inhibited H-cluster (Hox-CO). We investigated the reduced, CO-inhibited H-cluster (Hred´-CO) in this work. The stretching vibrations of the diatomic ligands were monitored by attenuated total reflection Fourier-transform infrared spectroscopy (ATR FTIR). Density functional theory (DFT) at the TPSSh/TZVP level was employed to analyze the cofactor geometry, as well as the redox and protonation state of the H-cluster. Selective ^13^CO isotope editing, spectro-electrochemistry, and correlation analysis of IR data identified a one-electron reduced, protonated [4Fe–4S] cluster and an apical CN^−^ ligand at the diiron site in Hred´-CO. The reduced, CO-inhibited H-cluster forms independently of the sequence of CO binding and cofactor reduction, which implies that the ligand rearrangement at the diiron site upon CO inhibition is independent of the redox and protonation state of the [4Fe–4S] cluster. The relation of coordination dynamics to cofactor redox and protonation changes in hydrogen conversion catalysis and inhibition is discussed.

## 1. Introduction

Hydrogenases [1] are remarkably efficient catalysts for hydrogen conversion with significant potential in renewable energy applications [2,3,4]. The chemistry at the transition metal cofactor is based on a sophisticated interplay between redox and protonation changes, as well as protein–cofactor interactions [5,6,7] and is of prime interest for the design of biomimetic, synthetic hydrogen conversion catalysts [8,9,10]. Accordingly, hydrogenase proteins have been extensively characterized by X-ray crystallography [5], protein film electrochemistry [11,12,13], and numerous spectroscopic techniques [14]. However, the relations between coordination dynamics at the active site and redox chemistry are still under debate [15].

**Figure 1:**
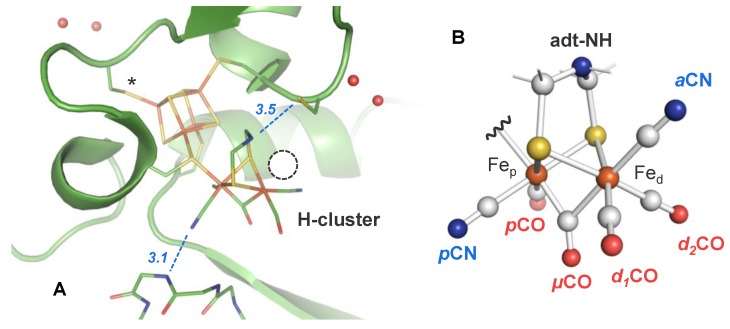
Crystal structure of the H-cluster in [FeFe]-hydrogenase. (**A**) The structure ( protein data bank entry 4XDC) [28] of the *Clostridium pasteurianum* enzyme (CPI) is poised in the oxidized resting state (Hox). The CN^‒^ ligand at Fe_p_ is stabilized by a hydrogen bond (blue dashes) to the protein backbone [29], whereas the diatomic ligands at Fe_d_ are located in a hydrophobic pocket. The aminodithiolate (adt) group may be hydrogen-bonded to C299 (blue dashes). The asterisk marks a likely protonation site at the [4Fe–4S] cluster. Catalytic protons are exchanged via adt-NH and C299 in the putative proton transfer pathway to the diiron site. The circle marks the open coordination site at Fe_d_ where exogenous CO initially binds to form the CO-inhibited, oxidized state (Hox-CO). (**B**) Proposed structure of the H-cluster in the Hox-CO state (the [4Fe–4S] cluster is omitted for clarity). The orientation of the diatomic ligands in various cofactor states is under debate.

[FeFe]-hydrogenases are found in archaea, bacteria, and unicellular algae [16]. They show truly bidirectional hydrogen conversion (i.e., catalysis of H_2_ oxidation and proton reduction at similar rates) [17]. In the cell, [FeFe]-hydrogenases typically serve as “electron valves” that prevent the accumulation of excess reducing equivalents by the release of H_2_, e.g., in photosynthetic algae such as *Chlamydomonas reinhardtii* [18]. Their unique catalytic cofactor, the so-called H-cluster, comprises a canonical [4Fe–4S] cluster linked by a cysteine thiolate to a diiron complex, [FeFe] [19,20]. The pendant amine base of an aminodithiolate group (adt-NH) serves as proton relay between the diiron site and the adjacent amino acid residues [21,22,23]. In the active-ready, oxidized state (Hox), the [FeFe] site binds two terminal carbon monoxide (CO) and cyanide (CN^‒^) ligands and a single carbonyl (*µ*CO) in the Fe–Fe bridging position (Figure 1). The natural presence of CO and CN^‒^ ligands at the active site of [FeFe]-hydrogenases facilitates cofactor-specific investigation by infrared spectroscopy [24,25,26,27].

Protein crystallography on oxidized [FeFe]-hydrogenases has shown that the diiron site adopts an unusual inverted-pyramid geometry with a *µ*CO ligand and an open coordination site at Fe_d_ (Figure 1A) [19,20]. In Hox, the H-cluster has been assigned to a formal [4Fe–4S]^2+^-[FeFe^I,II^] redox configuration [30]. Exogenous CO or O_2_ compete with H_2_ at the open coordination site of the oxidized cofactor [31]. However, while the reaction with O_2_ finally causes cofactor degradation, CO binding at the H-cluster in Hox induces the reversible formation of the CO-inhibited, oxidized state (Hox-CO) (Figure 1B) [31,32,33]. Little is known about CO binding to the reduced cofactor, which is the central topic of the present study.

The [FeFe]-hydrogenase from *C. reinhardtii* (HYDA1) is particularly well-suited for spectroscopic studies, because it exclusively binds the H-cluster and no further iron–sulfur clusters [34]. In recent work, we have extensively characterized HYDA1 protein films by attenuated total reflection Fourier-transform infrared spectroscopy (ATR FTIR) in combination with gas exposure, spectro-electrochemistry, and ^13^CO isotope editing [35,36,37,38,39,40]. The cofactor geometries and isotope editing patterns, as well as the redox and protonation states, were assigned by quantum chemical calculations (density functional theory, DFT). Previous work suggests that rearrangement of the ligands at [FeFe] relative to Hox is involved in the formation of Hox-CO and the reduced H-cluster species Hred and Hsred [35,36]. Furthermore, protonation at the [4Fe–4S] cluster in a one-electron reduced species with a *µ*CO ligand (Hred´) was implied [38,39]. These results tempted an assignment of H-cluster intermediates with a *µ*CO to the catalytic cycle of H_2_ conversion and of species lacking a *µ*CO to regulatory reactions [41]. The apparent mobility of the ligands at the distal iron seems to be crucial for H_2_ sensing and CO inhibition. However, whether CO binding affects the ligand geometry in the reduced H-cluster has not been addressed.

In this study, ^13^CO isotope editing and ATR FTIR spectro-electrochemistry on HYDA1 protein films was employed to poise the H-cluster in the CO-inhibited, oxidized state (Hox-CO) or one-electron reduced state (Hred´-CO). DFT facilitated an assignment of the experimental IR band patterns to underlying cofactor geometries. In comparison with Hox, our analysis suggests that Hred´-CO and Hox-CO show similar ligand reorientation at Fe_d_ with an apical CN^‒^ ligand rather than an apical CO ligand. The experimental and computational results indicate that Hred´-CO comprises a reduced and protonated [4Fe–4S] cluster, similar to its non-inhibited counterpart, Hred´ [39]. These findings imply that ligand rearrangement upon CO-inhibition is independent of the redox and protonation state of the [4Fe–4S] cluster. The importance of proton-coupled electron transfer to the [4Fe–4S] cluster for stabilization of the *µ*CO geometry is emphasized.

## 2. Results

### 2.1. ATR-FTIR Spectro-Electrochemistry and ^13^CO Isotope Editing

**Figure 2:**
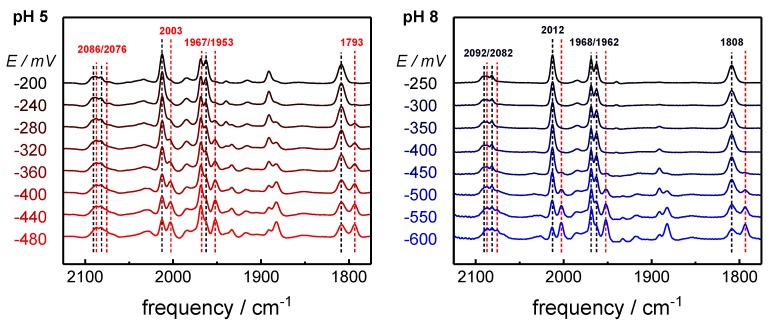
Formation of the one-electron reduced state, CO-inhinbited state Hred´-CO at pH 5 and pH 8. The spectra show the gradual population of Hred´-CO (red band frequency labels) at the expense of Hox-CO (black band frequency labels) for a step-wise decrease of redox potential at pH 5 (left panel) or pH 8 (right panel). Spectra were normalized to uniform integral band intensity. Minor bands are due to small reduced H-cluster species Hred and Hsred populations (e.g., at 1891 cm^−1^ and 1882 cm^−1^) and unrelated to the CO-inhibited states.

**Figure 3:**
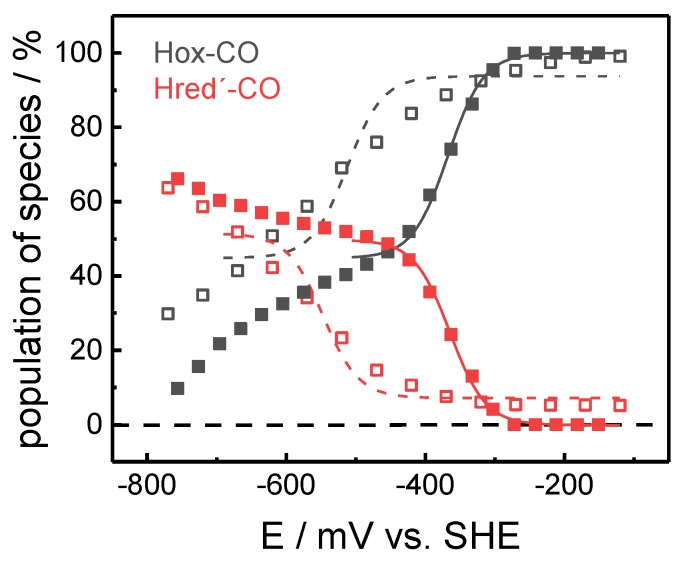
Hox-CO → Hred´-CO redox transition as function of potential. The conversion of Hox-CO (black symbols) to Hred´-CO (red symbols) was probed at pH 5 (solid symbols) or pH 8 (open symbols). The lines show fit curves using the Nernst equation. At pH 5 a midpoint potential (E_m_) of −360 ± 10 mV was determined, while at pH 8, E_m_ was shifted to −530 ± 30 mV. The larger error for the E_m_ value at pH 5 was due to the increasing formation of Hsred at the expense of Hred´-CO at low potentials, which caused further changes in the Hox-CO and Hred´-CO populations (compare Figure 2).

Purified [FeFe]-hydrogenase HYDA1 apo-protein was maturated *in vitro* with a synthetic diiron complex (Fe_2_(µ-adt)(CO)_4_(CN)_2_, adt = (SCH_2_)_2_NH) [42,43]. The catalytically competent enzyme was injected onto a thin gold mesh that was used to cover the silicon crystal of an ATR cell. This set-up facilitates both *in situ* FTIR spectro-electrochemistry and monitoring changes in the protein film upon exposure to varying atmospheres in the sample headspace, in particular ^13^CO isotope editing (see below). Increasingly reducing potentials (−100 to −800 mV vs. normal hydrogen electrode, NHE) were applied to CO-inhibited films of HYDA1 protein (Figure 2). In agreement with earlier studies, the reduction of the CO-inhibited cofactor was identified by a CO/CN^‒^ band pattern similar to Hox-CO but shifted by 5–10 cm^−1^ to lower frequencies [22]. Reminiscent of the spectral differences between Hox and Hred´ [39], the relatively small frequency shifts suggested that a one-electron reduced state with a similar cofactor geometry as in Hox-CO was formed, including a low-frequency *µ*CO band at 1793 cm^−1^. In the following, we will refer to this state as Hred´-CO. Previous studies have revealed that the summed IR band intensities of the oxidized and reduced H-cluster species can be considered constant [38,39]. Accordingly, the overall band intensity of each cofactor species corresponds to its fractional population in the sample. Determination of the population of states thus facilitated an assignment of the redox midpoint potential (E_m_) for the Hox-CO → Hred´-CO transition (Figure 3). Using the Nernst equation, this approach yielded E_m_ values of −365 ± 10 mV and −53 0 ± 30 mV versus NHE at pH 5 and pH 8, respectively (ΔE_m_ = 165 ± 30 mV).

**Figure 4:**
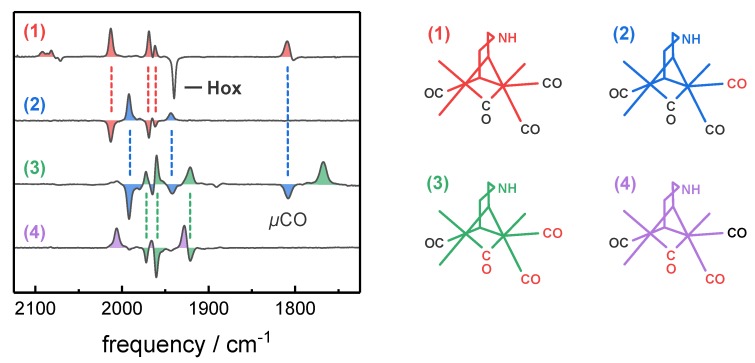
CO inhibition of Hox and preparation of Hox-CO isotopomers 1–4. Shown are *in situ* attenuated total reflection Fourier-transform infrared spectroscopy (ATR-FTIR) difference spectra of *Chlamydomonas reinhardtii* (HYDA1) in the CO/CN^‒^ frequency regime of the H-cluster (left panel) and corresponding diiron site structures with indicated ^13^CO labeling patterns (right panel, ^13^CO in red). Hox-CO species 1 is formed upon exposure of the oxidized enzyme (Hox) to ^12^CO gas in the dark. The exposure of isotopomer 1 to ^13^CO gas in the dark yields species 2, further illumination with white light under ^13^CO results in isotopomer 3. The exposure of isotopomer 3 to ^12^CO in the dark results in the formation of isotopomer 4. Note that the frequencies of the CN^‒^ ligands are barely affected by ^13^CO isotope editing, while the Hox → Hox-CO conversion is associated with difference signals in the CN^‒^ regime as well (2050–2100 cm^−1^).

Prior to isotope editing, the self-oxidation activity of HYDA1 was employed to accumulate Hox in the presence of N_2_. Thereafter, exposure to ^12^CO or ^13^CO gas in the dark or in combination with white light illumination resulted in the selective enrichment of Hox-CO isotopomers 1–4 with different ^13^CO labeling patterns (Figure 4), as reported previously [35]. The determined IR frequencies and intensities of the CO/CN^‒^ ligands at the H-cluster are listed in Table 1 and Table S1.

**Figure 5:**
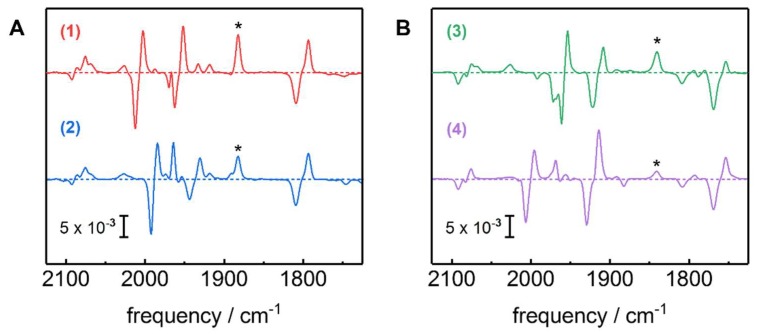
Hox-CO → Hred´-CO conversion for ^13^CO isotopomers 1–4. The *in situ* ATR-FTIR difference spectra of the H-cluster show Hox-CO at −200 mV (negative bands) and Hred´-CO at −800 mV versus normal hydrogen electrode (NHE, positive bands). Panel (**A**) shows isotopomers 1 and 2 with a *µ*^12^CO ligand, while in (**B**) the bridging ligand is exchanged to ^13^CO (isotopomers 3 and 4). * Bands due to Hsred populations.

**Figure 6:**
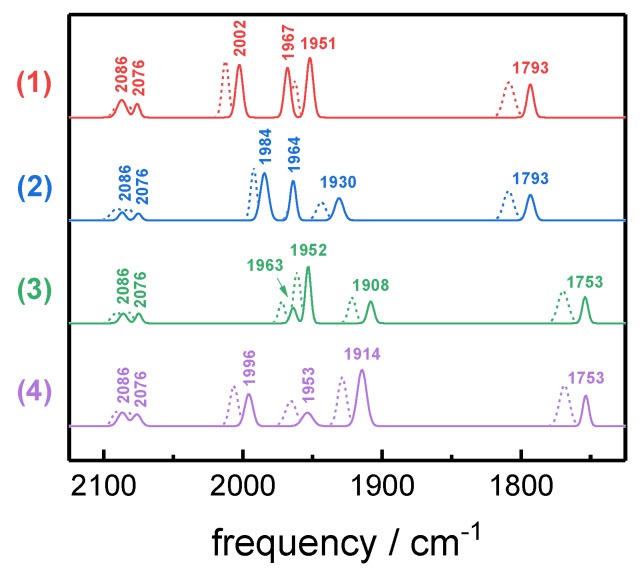
Pure IR spectra of Hred´-CO isotopomers 1–4. Spectro-electrochemical experiments yielded pure IR spectra for Hox-CO (dotted lines) and Hred´-CO (solid lines) after the subtraction of the minor IR contributions of other H-cluster species, such as Hred and Hsred, and the other isotopomers of Hox-CO/Hred´-CO (compare Figure 5). The CO/CN^−^ stretching frequencies for Hred´-CO are annotated.

Potential jump experiments were applied to convert Hox-CO ^13^CO isotopomers 1–4 into the corresponding Hred´-CO species. The resulting in situ difference spectra are shown in Figure 5. For all isotopomers, the Hox-CO → Hred´-CO transition was accompanied by downshifts of CO/CN^‒^ band frequencies of about 6–15 cm^−1^. Interestingly, the symmetrical stretching vibrations of the distal CO ligands (d_1_ and d_2_) in most isotopomers showed changes in band intensity besides frequency shifts. Minor populations of non-inhibited reduced states (i.e., Hred and Hsred) were subtracted to gain pure spectra of Hox-CO and Hred´-CO (Figure 6). The IR frequencies and intensities of the four Hred´-CO isotopomers are compiled in Table 1 and Table S1.

### 2.2. CO binding in the Presence of H_2_

**Figure 7:**
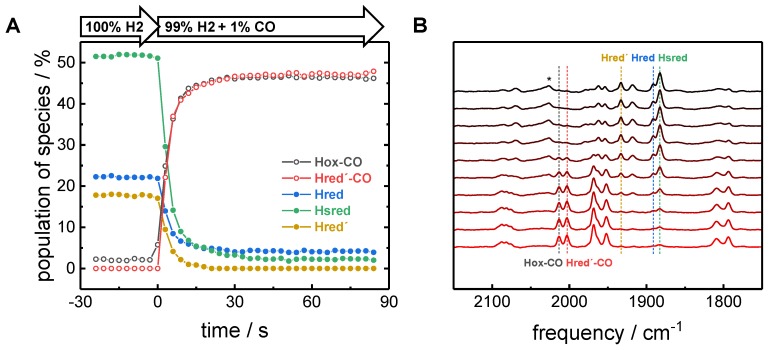
CO inhibition of pre-reduced HYDA1. Kinetic traces in (**A**) and corresponding FTIR spectra in (**B**). [FeFe]-hydrogenase was first exposed to 100% H_2_ to accumulate the reduced species Hred, Hsred, and Hred´. No external potential was applied. Upon injection of 1% CO into the gas stream, the H-cluster rapidly forms about equal populations of Hox-CO and Hred´-CO. While Hred´ is lost within ~10 s, Hred and Hsred decrease to reach a stable population of around 5% after ~1 min. * The high-frequency band at 2026 cm^−1^ has been to assigned to Hsred and is unrelated to the CO-inhibited species.

Our results clearly indicate the enrichment of Hred´-CO in films where HYDA1 was first inhibited by exogenous CO and reduced afterwards. In order to address the question whether CO binding to the reduced H-cluster results in alternative CO-inhibited species, HYDA1 was first reduced with H_2_ (open circuit potential) and thereafter exposed to a CO atmosphere (1% CO in 99% H_2_). This approach resulted in the concomitant increase of Hox-CO and Hred´-CO in the film, but further CO-inhibited species were not observed (Figure 7). The complete loss of Hred´ within less than 10 s after addition of CO resembles the complete loss of Hox under N_2_. However, small fractions of Hred and Hsred remained detectable even after several minutes of CO exposure. This hints at differences in CO sensitivity between Hred´ and Hred/Hsred.

### 2.3. Assignment of H-Cluster Species by Density Functional Theory

**Figure 8:**
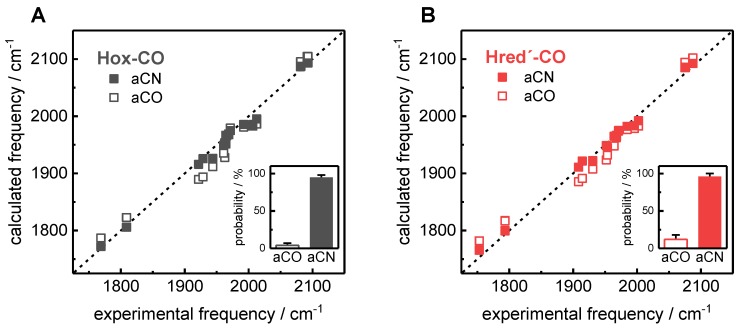
Correlation analysis for Hox-CO and Hred´-CO. The calculated IR frequencies stem from density functional theory (DFT) calculations (corrected for systematic deviations; Equations (1) and (2); Figures S1 and S2, Tables S1–S3). H-cluster structures: apical CO at Fe_d_ (aCO, open symbols), apical CN^‒^ at Fe_d_ (aCN, solid symbols). The lines show the diagonal for ideal correlation ((**A**), Hox-CO: mean rmsd of 20 cm^−1^ for aCO or 8 cm^−1^ for aCN. (**B**), Hred´-CO (including a proton at the [4Fe–4S] cluster): mean rmsd of 19 cm^−1^ for aCO or 7 cm^−1^ for aCN, Table S3). Insets: apparent probability of aCO and aCN structures (Table S4).

DFT was employed to generate geometry-optimized H-cluster model structures and to calculate CO/CN^‒^ vibrational frequencies by normal mode analysis, as previously reported (whole cofactor structures, TPSSh/TZVP functional/basis-set combination) [35]. Correlation analysis of experimental and calculated IR frequencies and intensities was carried out (Equations (1)–(3)). This procedure revealed that the IR spectra of Hox-CO and Hred´-CO for the four ^13^CO isotopomers were much better described by H-cluster structures with an apical cyanide ligand (aCN) instead of an apical carbonyl ligand (aCO) at Fe_d_ (Figure 8, Figures S1 and S2; Tables S1–S4). Linear regressions of calculated versus experimental data consistently revealed about two-fold smaller errors of offset and slope parameters and a larger R^2^ value, which indicates a significantly better fit quality for the aCN structures. The root-mean-square deviation (rmsd) for the calculated IR frequencies (corrected for systematic theory-inherent deviations) for all ^13^CO patterns with an aCN ligand was about three-fold smaller (mean of ~7 cm^−1^ vs. ~20 cm^−1^). For the band intensities, about two-fold smaller rmsd values were observed for the aCN structures (mean of ~7% vs. ~13%). The computational results thus facilitated a unique assignment of the ^13^CO labeling patterns in the four isotopomers of Hox-CO and Hred´-CO (Table 1). We have previously assigned protonation at a sulfur atom of a cysteine ligand (C417 in HYDA1) of the [4Fe–4S] cluster in Hred´ [39]. A comparison of structures with or without such a proton at the [4Fe–4S] cluster resulted in a significantly improved match between the calculated and experimental IR data for Hred´-CO. The calculation of apparent relative probabilities of H-cluster structures from the R^2^ and rmsd values from the IR data correlations further supported an apical CN^−^ in Hox-CO and Hred´-CO and a surplus proton at the [4Fe–4S] cluster in Hred´-CO (Figure 8).

## 3. Discussion

FTIR spectro-electrochemistry and quantum chemical calculations have resulted in a conclusive characterization of the reduced, CO-inhibited state Hred´-CO. We report the full vibrational spectrum of the H-cluster including the CO and CN^–^ bands of four ^13^CO labeled isotopomers of Hred´-CO while previously only CO stretching frequencies were reported [22]. Four isotopomers were generated by selective ^13^CO isotope editing [35] and clearly assigned by the computational results. Furthermore, we monitored the Hox-CO → Hred´-CO conversion as a function of both redox potential and pH. The decrease of the redox midpoint potential by about 60 mV per pH unit indicates that the formation of Hred´-CO is accompanied by protonation of the H-cluster. This is reminiscent of the Hox → Hred´ transition for which ATR FTIR spectro-electrochemistry and DFT data have identified a cysteine ligand at the [4Fe–4S] cluster as most likely protonation site in Hred´ [38,39]. Our present computational results suggest a similar protonation in Hred´-CO and support the tentative assignment of a one-electron reduced [4Fe–4S] cluster [22]. Accordingly, the presence of an additional CO ligand at the diiron site seems not to affect the protonation/reduction behavior of the [4Fe–4S] cluster. We have proposed earlier that the proton stabilizes a reduced [4Fe–4S] cluster in Hred´ and prevents formation of Hred and Hsred with a reduced diiron site [41]. Carbon monoxide is expected to bind preferably at the oxidized diiron site [17], which agrees with the observation that only Hred´-CO and no CO-inhibited species with a reduced diiron site are observed.

Our analyses clearly favor an apical CN^‒^ at Fe_d_ in Hox-CO and Hred´-CO. The crystal structure of CO-inhibited [FeFe]-hydrogenase from *Clostridium pasteurianum* (CPI) was interpreted to bind exogenous CO in an apical orientation at Fe_d_ [19,32]. Furthermore, changes in the electron density distribution between dark-adapted and illuminated CPI crystals apparently supported this view, however also showed differences at various other positions of the cofactor (e.g., a nearby methionine and all around Fe_d_) [44]. The limited resolution of the protein crystallography prohibits a unique distinction between CO and CN^‒^. Therefore, the crystallographic assignment of cofactor geometry in the CO-inhibited state remains ambiguous. We find that an apical CN^‒^ describes the IR-band frequency and intensity patterns for all 16 possible ^13^CO isotopomers of Hox-CO and four ^13^CO isotopomers of Hred´-CO consistently better than an apical CO [35]. The secondary amine of the dithiolate ligand at the diiron site (adt-NH) may form a hydrogen bond with an apical, negatively charged cyanide, thereby stabilizing the CO-inhibited cofactor. Intramolecular stabilization between adt and the apical ligand has been suggested for Hhyd [37] and previously. Electrostatic attraction is not expected with a carbonyl ligand and can explain the pronounced CO sensitivity of [FeFe]-hydrogenases. The absence of hydrogen bonding to CN^‒^ may be part of the reason why HYDA1 with a pdt ((SCH_2_)_2_CH_2_) instead of an adt dithiolate is apparently not inhibited by CO [22,45].

Our findings imply that diatomic ligand rearrangement accompanies Hox-CO and Hred´-CO formation. The reduction of the H-cluster after CO inhibition (i.e., starting from Hox-CO) readily results in Hred´-CO and no further CO/CN^‒^ reorientation needs to take place. However, when the H-cluster was first poised in Hred´ and exposed to CO thereafter, only Hred´-CO and Hox-CO and no structural isomers of the CO-inhibited species were formed. CO/CN^‒^ ligand rearrangement, hence, is independent of the redox and protonation state of the [4Fe–4S] cluster and primarily governed by the structural and electronic properties of the diiron site, as suggested earlier [15]. This conclusion is corroborated by the observation that small yet significant fractions of Hred/Hsred remain detectable after prolonged CO exposure. In these states, CO binding and ligand rearrangement may be retarded due to the stabilization of an altered diiron site geometry with an apical CO ligand [36,46], whereas redox species with a vacant Fe_d_ site such as Hox and Hred´ react instantaneously with CO [17]. We note that the ligand rearrangement at Fe_d_ was questioned due to a potential salt bridge of the distal CN^‒^ to a lysine residue in the active site niche [47,48,49]. However, at room temperature, such an interaction may be overcome by the vibrational dynamics of the cofactor–protein system [15], finally resulting in hydrogen-bonding between CN^‒^ and adt-NH in the CO-inhibited states.

In conclusion, the H-cluster appears to be optimized to prevent ligand rearrangement at the diiron site during rapid H_2_ conversion [41]. The stabilization of a cofactor geometry with a *µ*CO ligand in the catalytic cycle is achieved by site-selective reduction and protonation at the [4Fe–4S] cluster in the first redox step. Preventing ligand rearrangement, for example, in enzymes with tailored cofactor variants, may improve the CO tolerance of [FeFe]-hydrogenases in hydrogen fuel production applications.

## 4. Materials and Methods

### 4.1. Sample Preparation

[FeFe]-hydrogenase HYDA1 from *Chlamydomonas reinhardtii* was heterologously expressed in *E. coli*, isolated as an apo-protein (including only the [4Fe–4S] cluster), and activated *in vitro* with an adt-NH containing a synthetic diiron complex as previously described [42,43].

### 4.2. Fourier-Transform Infrared Spectroscopy

Infrared spectroscopy was conducted at room temperature (24 °C) inside an anaerobic chamber (Coy, less than 2 ppm O_2_) on a FTIR spectrometer (Tensor27, Bruker, Esslingen, Germany) equipped with a HgCdTe photodiode (Kolmar Technologies, Newburyport, MA, USA) and an ATR unit (DuraSamplIR II, Smiths Detection, London, UK). The stainless-steel ATR crystal plate was covered with non-conductive Kapton tape, leaving a hole for the Si crystal. A small strip of 9 μm thick Au mesh was deposited on the Si crystal, and 1 μL of HYDA1 protein (about 500 μM) was pipetted onto the Si crystal. ATR-FTIR spectra were recorded with a spectral resolution of 2 cm^−1^ and various co-additions of interferometer scans (a typical time resolution of ~5 s was achieved with 25 scans). Further details on the experimental set-up, data processing, and evaluation procedures (including baseline subtraction and spectral fit approaches) can be found in [38,39].

Before spectro-electrochemistry, ^13^CO isotope editing was performed to accumulate Hox-CO isotopomers 1–4, as previously reported [35,36]. Species 1 shows no ^13^CO ligands and is formed upon contact of oxidized protein (Hox) with ^12^CO gas. Species 2 carries a single ^13^CO ligand at Fe_d_ and accumulates in the presence of ^13^CO gas in the dark. For Species 3, the sample film is additionally irradiated with white light, which allows the exchanging of both CO ligands at Fe_d_ to ^13^CO, as well as *µ*CO. Species 4 is formed upon contact of Species 3 with ^12^CO in the dark. Note that in all the analyzed isotopomers, Fe_p_-CO remains unchanged (Figure 2).

After isotope editing, a patch of a dialysis membrane (8 kDa cut-off) was positioned on the Au-embedded protein film to prevent dilution of the protein sample. A gas-tight polychlorotrifluoroethylene (PCTFE) cell was screwed onto the crystal plate. The reaction cell comprises a copper ring on its lower surface for contacting the Au working electrode through the hole in the membrane. The upper reservoir of the reaction chamber was filled with 3 mL of electrolyte buffer (e.g., 100 mM MES or Tris/HCl, 100 mM KCl, pH 5 or 8) and equipped with a Pt counter electrode and an Ag/AgCl reference electrode. No redox mediators were used. To populate various redox states of the H-cluster, redox potentials in the range of about −100 to −800 mV versus NHE were adjusted by a potentiostat (PARSTAT 2273, Ametek Scientific Instruments, Columbus, OH, USA), and IR spectra were recorded at increasing time periods after switching the potential.

### 4.3. Density Functional Theory

DFT was carried out using the Gaussian09 program [50] and the TPSSh/TZVP functional/basis-set combination [51,52,53]. H-cluster model structures included the whole cofactor with truncated cysteine residues saturated with protons as described earlier [35]. Compared to Hox-CO, Hred´-CO structures were reduced by one electron (localized at the [4Fe–4S] cluster), excluding or including an additional proton at the sulfur atom of the C417 ligand of the [4Fe–4S] cluster [39]. Unrestrained geometry optimization on the H-cluster models, including varying ^13^CO labeling patterns and either an apical CO ligand at Fe_d_ (aCO) or an apical CN^‒^ ligand (and two equatorial CO ligands) at Fe_d_ (aCN), was followed by normal mode analysis to derive IR frequencies and intensities of the CO/CN^‒^ ligands. The calculated and experimental IR intensities were normalized to a sum of 100% over all CO/CN^‒^ ligands for comparison [35]. Prior to calculation of the root-mean-square deviation (rmsd) according to Equation (2) between experimental and calculated IR frequencies and intensities, the calculated data (F,I_cal_) was corrected (F,I_cor_) for theory-inherent deviations from ideal correlation with experimental data using the results of linear regression analysis (respective offset and slope values; Figures S1 and S2 and Tables S1 and S2) and Equation (1) for the alignment of the experimental and calculated IR spectra.

F,I_cor_ = (F,I_cal_ − offset_F,I_)/slope_F,I_ (1)

(2) $$\mathrm{rmsd}=\sqrt{\sum_{n}{\left({F,I}_{\mathrm{cor}}-{F,I}_{\exp}\right)}^{2}/n}.$$

Rmsd values are listed in Table S3. Apparent relative probabilities (*P*, in %) of H-cluster structures (Table S4, Figure 8) were calculated from R^2^ and rmsd values of IR data correlations for Hox-CO and Hred´-CO species using Equation (3) (min/max = minimal/maximal R^2^ or rmsd values for frequencies/intensities (i) within the series of protonated or unprotonated, aCN or aCO structures):

(3) $$P=100\sum_{i-n}\left[\left(\frac{{R_{i}}^{2}-{{R_{i}}^{2}}_{\min}}{{{R_{i}}^{2}}_{\max}-{{R_{i}}^{2}}_{\min}}\right)+\left(\frac{{\mathrm{rmsd}}_{i}-{\mathrm{rmsd}}_{i\max}}{{\mathrm{rmsd}}_{i\min}-{\mathrm{rmsd}}_{i\max}}\right)\right]/n.$$

## Figures and Tables

**Table 1:** CO/CN^‒^ stretching frequencies of CO-inhibited H-cluster isotopomers ^a^.

H-Cluster Species		CO Pattern ^a^	Vibrational Frequency (cm^−1^)					
		p µ d_1_ d_2_	CN^‒^		CO			*µ*CO
**Hox-CO**	1	12 12 12 12	2092	2082	2012	1968	1962	1808
	2	12 12 12 13	2092	2082	1991	1964	1942	1808
	3	12 13 13 13	2092	2082	1972	1960	1920	1768
	4	12 13 13 12	2092	2082	2006	1964	1927	1768
**Hred´-CO**	1	12 12 12 12	2086	2076	2002	1967	1951	1793
	2	12 12 12 13	2086	2076	1984	1964	1930	1793
	3	12 13 13 13	2086	2076	1964	1952	1908	1753
	4	12 13 13 12	2086	2076	1996	1954	1914	1753

^a^ Position of CO ligands at [FeFe]: p, proximal Fe; µ, Fe–Fe bridging; d, distal Fe (1 and 2). Ligand position and isotopic labeling assignments are based on experimental and computational evidence in the present study and earlier reports (compare Figure 2) [35].

## Supplementary Materials

Supplementary materials are available on line.
