# Supplementary material for: Spectroscopical Investigations on the Redox Chemistry of [FeFe]-Hydrogenases in the Presence of Carbon Monoxide
Source: Molecules. 2018 Jul 9;23(7):1669. doi: 10.3390/molecules23071669 (PMC6100070; doi:10.3390/molecules23071669)
Supplement: Supplementary file 1 [file molecules-23-01669-s001.pdf]

Supporting Information

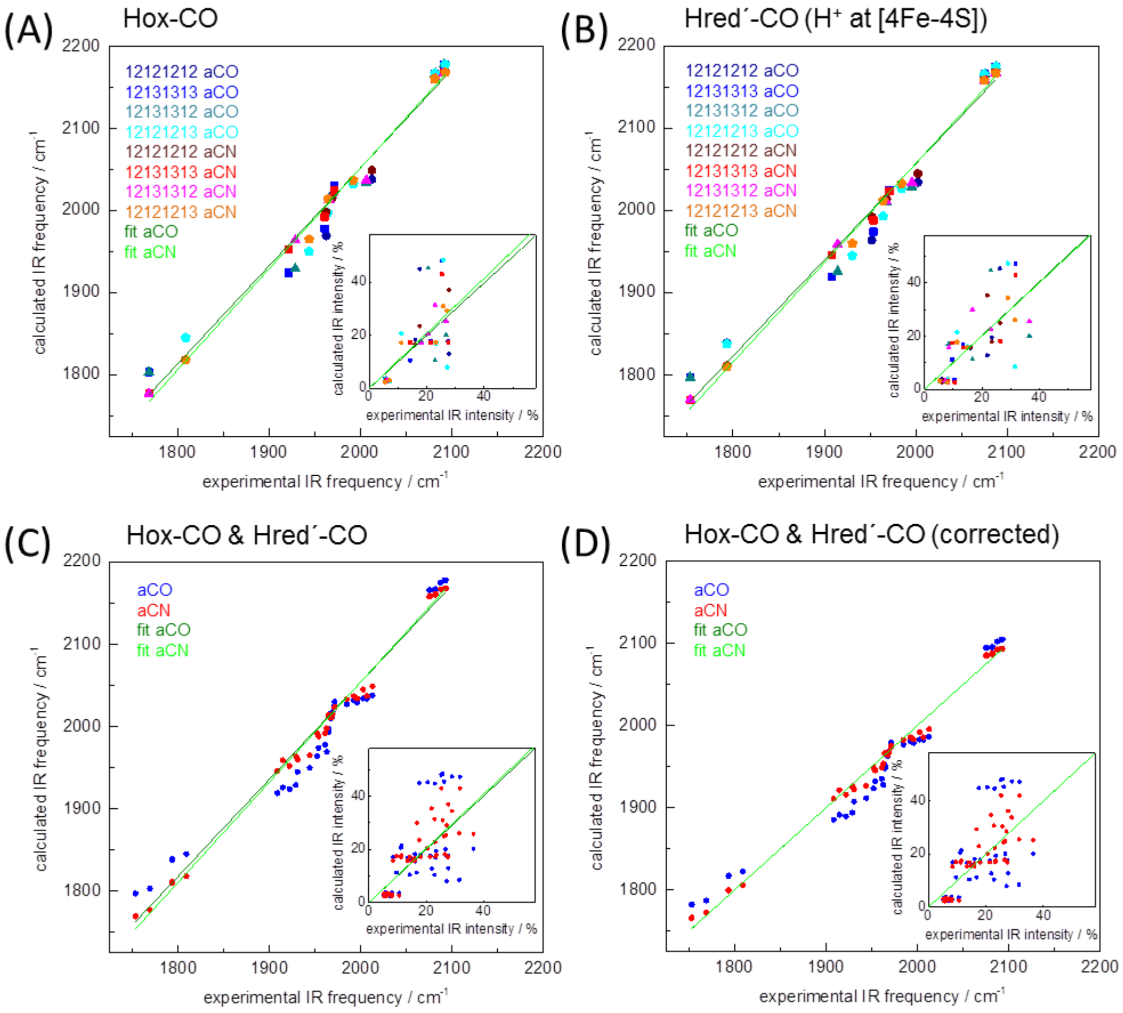

Figure S1 - Correlations of experimental and calculated IR data. DFT models: aCO, apical CO ligand at Fe$_d$; aCN, apical CN ligand at Fe$_d$. Data stem from Table S1 for the indicated CO labeling patterns of the H-cluster (p µ d1 d2), lines show linear regressions with parameters in Table S2 for fits of IR frequency and intensity (inset) data of the CO/CN ligands for Hox-CO (A), Hred´-CO (including a proton at the [4Fe-4S] cluster) (B), or all CO labeling data of both states (C). In (D), DFT data corrected (Eq. 1) for systematic deviations from ideal correlation (i.e. offset = 0, slope = 1) are shown for all CO labeling data of both states.

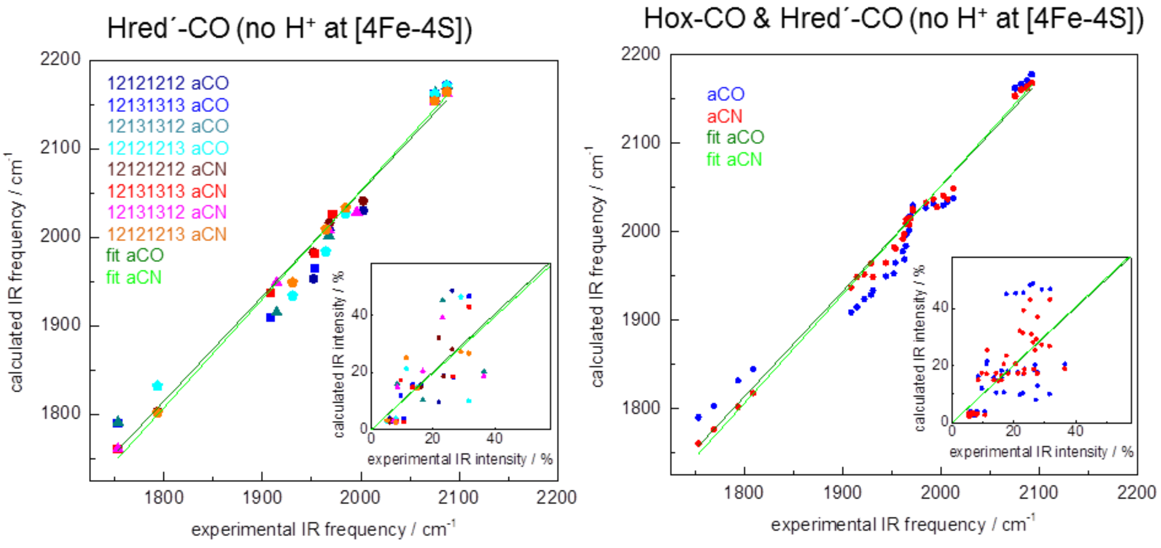

Figure S2 - Correlations for Hred´-CO without [4Fe-4S] cluster protonation. Annotations as in Fig. S3. The proton at the [4Fe-4S] cluster in Hred´-CO was omitted in the structures.

Table S1: Experimental and calculated IR frequencies and intensities.[a]

state	ligand	EXP				DFT							
						aCO model structure				aCN model structure			
		CO labeling pattern (p µ d1 d2)											
		12121212	12121213	12131313	12131312	12121212	12121213	12131313	12131312	12121212	12121213	12131313	12131312
		IR frequency [cm^{-1}]											
	CO	1808	1808	1768	1768	1845	1845	1803	1803	1818	1818	1777	1777
		1962	1942	1920	1927	1969	1929	1924	1950	1998	1965	1952	1965
		1968	1964	1960	1964	2018	2015	1978	1997	2015	2013	1992	2014
		2012	1991	1972	2006	2038	2035	2030	2031	2049	2038	2024	2036
	CN	2082	2082	2082	2082	2167	2167	2167	2167	2161	2161	2160	2161
		2092	2092	2092	2092	2178	2178	2178	2178	2168	2168	2168	2168
Hox-CO		normalized IR intensity [%]											
	CO	29	25	28	25	18	18	18	17	17	17	17	17
		16	15	16	26	18	20	18	20	17	25	17	17
		17	15	30	16	45	45	48	49	23	20	43	31
		28	27	11	20	13	11	10	8	37	32	17	29
	CN	4	7	6	6	3	3	3	3	3	3	3	3
		5	12	9	7	4	4	3	4	2	2	2	2
		IR frequency [cm^{-1}]											
	CO	1793	1793	1753	1753	1839	1839	1797	1797	1811	1810	1769	1770
		1951	1930	1906	1914	1964	1926	1919	1945	1992	1959	1946	1960
		1967	1962	1953	1962	2013	2010	1974	1993	2014	2011	1988	2012
		2002	1984	1964	1996	2034	2030	2025	2026	2045	2035	2023	2033
	CN	2076	2076	2076	2076	2166	2166	2166	2166	2159	2158	2158	2158
Hred´-CO		2086	2086	2086	2086	2175	2175	2175	2175	2167	2167	2167	2167
($+H^+$)		normalized IR intensity [%]											
	CO	14	17	19	14	16	17	17	16	16	16	16	16
		26	17	17	39	19	20	18	21	18	26	18	18
		21	21	38	11	45	44	47	48	25	23	43	35
		25	35	11	20	13	12	11	8	35	30	17	26
	CN	5	4	7	7	3	3	3	3	3	3	3	3
		9	5	9	9	4	4	4	4	3	3	3	3
		IR frequency [cm^{-1}]											
	CO	1793	1793	1753	1753	1832	1832	1790	1786	1803	1802	1761	1761
		1951	1930	1906	1914	1953	1915	1909	1930	1983	1951	1937	1949
		1967	1962	1953	1962	2008	2003	1965	1979	2016	2009	1981	2009
		2002	1984	1964	1996	2030	2029	2026	2017	2041	2031	2026	2032
	CN	2076	2076	2076	2076	2162	2162	2162	2163	2154	2154	2154	2154
Hred´-CO		2086	2086	2086	2086	2171	2171	2171	2168	2164	2164	2164	2164
($-H^+$)		normalized IR intensity [%]											
	CO	14	17	19	14	16	16	16	16	15	15	15	15
		26	17	17	39	19	20	18	20	19	19	19	19
		21	21	38	11	49	46	47	46	28	40	43	40
		25	35	11	20	10	11	12	11	32	21	18	21
	CN	5	4	7	7	3	3	3	3	3	3	3	3
		9	5	9	9	4	4	4	4	3	3	3	3

[a] For Hred´-CO, structures with ($+H^+$) or without ($-H^+$) a proton at the [4Fe-4S] cluster were considered.

Table S2: Fit parameters of IR frequency and intensity correlations.[a]

state	parameter	DFT structure					
		aCO			aCN		
		offset [cm^{-1}]	slope	R^2	offset [cm^{-1}]	slope	R^2
Hox-CO	IR frequency	-338(96)	1.20(5)	0.964	-409(38)	1.22(2)	0.995
Hred´-CO (+H^+)		-283(91)	1.17(5)	0.965	-361(35)	1.21(2)	0.995
Hred´-CO (-H^+)		-314(98)	1.18(6)	0.961	-398(37)	1.23(2)	0.994
		offset [%]	slope	R^2	offset [%]	slope	R^2
Hox-CO	IR intensity	0	0.99(14)	0.319	0	1.04(7)	0.728
Hred´-CO (+H^+)		0	1.01(12)	0.461	0	1.00(7)	0.667
Hred´-CO (-H^+)		0	1.01(13)	0.457	0	0.99(9)	0.583
		offset [cm^{-1}]	slope	R^2	offset [cm^{-1}]	slope	R^2
Hox-CO & Hred´-CO (+H^+)	IR frequency	-307(66)	1.18(4)	0.964	-382(27)	1.21(2)	0.995
Hox-CO & Hred´-CO (-H^+)		-326(68)	1.19(4)	0.963	-403(26)	1.23(2)	0.994
		offset [%]	slope	R^2	offset [%]	slope	R^2
Hox-CO & Hred´-CO (+H^+)	IR intensity	0	1.00(9)	0.388	0	1.02(5)	0.697
Hox-CO & Hred´-CO (-H^+)		0	1.00(10)	0.389	0	1.01(5)	0.654
		offset [cm^{-1}]	slope	R^2	offset [cm^{-1}]	slope	R^2
Hred´-CO (+H^+) – Hox-CO	IR frequency difference	-2.5(6)	0.11(6)	0.124	-0.4(4)	0.32(4)	0.781
Hred´-CO (-H^+) – Hox-CO		-4.0(1.1)	0.21(11)	0.106	0.3(7)	0.66(8)	0.755

[a]Data refer to linear regressions ($F,I_{cal} = offset_{F,I} + slope_{F,I} \times F,I_{exp}$) to data points in Figs. S1 and S2 (aCO/aCN, apical CO or CN ligand at Fe_d). Annotations +/-H^+ for Hred´-CO refer to the presence or absence of a proton at the [4Fe-4S] cluster. Values for IR frequency differences refer to corrected data (Eq. 1). Values for experimental and computational IR frequency differences were derived from data in Table S1 and shown values refer to linear regression analysis.

Table S3: Root-mean square deviations for IR data of **Hox-CO** and **Hred´-CO**.[a]

state	model structure	parameter	CO labeling pattern (p µ d1 d2)				
			12 12 12 12	12 12 12 13	12 13 13 13	12 13 13 12	mean
Hox-CO			rmsd [cm^{-1}]				
	aCO	IR frequency	20	18	20	20	20
	aCN		8	8	7	9	8
			rmsd [%]				
	aCO	IR intensity	13	13	10	12	12
	aCN		6	5	9	4	6
Hred´-CO (+H$^+$)			rmsd [cm^{-1}]				
	aCO	IR frequency	20	18	20	19	19
	aCN		7	6	8	8	7
			rmsd [%]				
	aCO	IR intensity	9	13	8	12	11
	aCN		6	5	8	8	7
Hred´-CO (-H$^+$)			rmsd [cm^{-1}]				
	aCO	IR frequency	21	20	22	20	21
	aCN		8	7	8	8	8
			rmsd [%]				
	aCO	IR intensity	11	12	8	12	11
	aCN		5	6	7	11	7

[a]rmsd was calculated (Eq. 2) from experimental IR frequencies and normalized intensities (Table S1) and IR data from DFT corrected for systematic deviations (F/I$_{cor}$, Eq. 1). Data for DFT models with an apical CO (aCO) or CN (aCN) at Fe$_d$ are compared (+/-H$^+$, with/without H$^+$ at [4Fe-4S]).

Table S4: Relative probability of H-cluster structures.[a]

state	structure	
	aCO	aCN
	relative probability [%]	
Hox-CO	3±2	97±2
Hred´-CO (+H$^+$)	12±6	96±4
Hred´-CO (-H$^+$)	7±7	86±6

[a]Probabilities (±standard deviation) were calculated using Eq. 3 and represent the mean values for respective R^2 (from IR frequency and intensity correlations; Figs. S1 and S2) and rmsd (Table S3) values.
